# Better be prepared: the spectrum of neuropsychiatric impairment among Libyan war victims transferred to Germany for trauma rehabilitation

**DOI:** 10.1186/s42466-021-00134-z

**Published:** 2021-07-05

**Authors:** Felix Dootz, Otto-Magnus von Stackelberg, Joan Abaya, Christian Jacobi, Christoph Mohs, Eva Maria Craemer, Christoph Rangger, Uta Meyding-Lamadé, Eva Kathrin Lamadé

**Affiliations:** 1 BG Trauma Center Ludwigshafen, University of Heidelberg, Ludwigshafen am Rhein, Germany; 2 Department of Neurology, Krankenhaus Nordwest, Frankfurt/Main, Germany; 3 Ludwig-Maximilians-Universität, Munich, Germany; 4 International Institute of Medicine and Telemedicine, Frankfurt/Main, Germany; 5 Orthopedics and Trauma Surgery, Optimum Orthopädie, Frankfurt/Main, Germany

**Keywords:** War injuries, Neurology, Trauma surgery, Psychiatry, Interdisciplinary, Injury patterns

## Abstract

**Background:**

The current Libyan civil war has originated many casualties, imposing medical challenges. War injuries are complex, requiring specialized knowledge and interdisciplinary assessment for adequate patient and intercultural management.

**Methods:**

This retrospective study analyzed records of 78 Libyan patients admitted from July 2016 to November 2017 to neurological and trauma surgical departments of Krankenhaus Nordwest, Frankfurt, Germany. Issues of system preparation of the hospital, demographics, injury patterns and therapies were analyzed. The chi-squared test was used to analyze differences in injury patterns in explosion and gunshot injuries.

**Results:**

Seventy-seven of seventy-eight patients were male (mean age 30.6 years). The patients received primary and secondary treatment in Tunisia (*n* = 39), Libya (*n* = 36) and Turkey (*n* = 23). Forty-eight patients had gunshot injuries, 37 explosion injuries, 11 both. Preparation for management of injuries included hygienic and isolation protocols, organization of interpreters and intercultural training. Patients presented with a broad variety of neurological, psychiatric and trauma surgical injuries. Fifty-six patients had sensory, 47 motor deficits. Nine reported headache, 5 vertigo, 13 visual impairment, 28 psychiatric symptoms. Eighteen patients had central nervous damage, 50 peripheral nervous damage. Central nervous damage was significantly more common in gunshot than explosion injuries (*p* = 0.015). Peripheral nervous damage was more common in explosion than gunshot injuries (*p* < 0.1). Fifty-one patients had polytrauma and 49 suffered from fractures. Therapy included surgical interventions (*n* = 56) and physiotherapy. Structured rehabilitation programs were often indicated.

**Conclusion:**

Specialized knowledge about war injuries and their management including hospital preparation and planning regarding infrastructure may be required anytime. Injuries include a broad variety of neurological, psychiatric and trauma surgical injuries. Therefore, an interdisciplinary approach is crucial.

## Background

The current civil war in Libya is originating high numbers of casualties, including both civilian and military casualties. From 2012 to 2017, over 16.000 deaths and over 40.000 injured were reported, with injury and mortality rates peaking between 2015 and 2017 [12]. From April 2019 to April 2020, UNSMIL (United Nations Supporting Mission Libya) documented at least 685 civilian casualties [36]. This war is not only a humanitarian disaster but is also a great medical challenge. Several countries around the world are admitting war casualties for secondary and tertiary care, as on-site health facilities are often insufficient and destroyed by military action.

Thankfully, there is currently no war exposure in Germany. However, its onset is often unpredictable. Therefore, knowledge concerning preparation and management of war casualties, demographics, adequate diagnosis, disease patterns and treatment may be needed any time. However, literature covering an overview of interdisciplinary injury patterns is rare. War injuries predominantly affect the young male population and definite treatment (secondary and tertiary care) often occurs in neighboring countries [2, 3]. The mechanism of injury differs depending on type of warfare, weapons and military protective equipment. Civil wars affect civilians [25] who are without adequate armored protection. Explosion injuries, also called blast injuries, gunshots and burning injuries consequently have a worse effect with higher mortality among civilians [10, 23]. In recent civil wars, gunshot and explosion injuries have been reported as main mechanism of injury. Car accidents also occur [26].

Many trauma centers worldwide have plans for multi-cultural populations who encounter a mass casualty incident such as natural disasters, plane crashes, terrorism events, refugees and war, but many other hospitals may need to implement this care depending on the scope of the event. Key considerations for hospitals include training and education of staff, triage, rumor control [16] as well as hygienic protocols including decontamination protocols [30]. Krankenhaus Nordwest is a certified regional Trauma Center and is prepared for mass casualty incidents.

Neurological, psychiatric, orthopedic, trauma surgical expertise and specialized diagnostics such as computed tomography scans (CT), magnetic resonance imaging (MRI), electrophysiology and ultrasound examination are essential for adequate management of the injured [6, 15]. These are frequently not available in war zones. War injuries often affect multiple organs, as bullets and splinters transfer a high amount of kinetic energy to tissues, causing severe damage. They transmit energy to bone fragments, which themselves act as secondary bullets and increase damage to the surrounding tissue [3, 37]. This can frequently cause polytrauma and extensive neurological damage to the peripheral and central nervous system. Psychiatric comorbidities such as post-traumatic stress disorder are commonly detected in people with war experience [8, 27] and are known to have adverse effects on patients’ health such as an increased risk for homelessness [35] and can even affect the next generation [5]. Commonly diagnosed orthopedic injuries include complicated fractures, soft tissue damage and wound infections with multidrug resistant organisms (MDRO), which are described in up to 60% of cases [22]. The mortality rate of war victims is variable and is up to 31% [3]. Patients often have long-term damage and extended recovery periods after war incidents [7].

The aim of this retrospective analysis was to report structural preparation for management of war injuries and to analyze demographics, injury mechanisms, symptoms, diagnostics, injury patterns and therapies of 78 patients with war injuries that were treated in the departments of neurology and trauma surgery of Krankenhaus Nordwest, Frankfurt am Main (Germany) from July 2016 to November 2017.

## Methods

This study was conducted as a retrospective analysis. As part of an international cooperation between Libya and Krankenhaus Nordwest, 78 patients with war injuries were treated in Krankenhaus Nordwest from July 2016 to November 2017. All medical records were analyzed for issues of system preparations that enabled our hospital to care for the patients, for demographics, injury mechanisms, symptoms, diagnostics, neurological, psychiatric and trauma surgical injury patterns. All data from existing previous medical records of pretreatment and medical records from Krankenhaus Nordwest were included in the study.

**Fig. 1:**
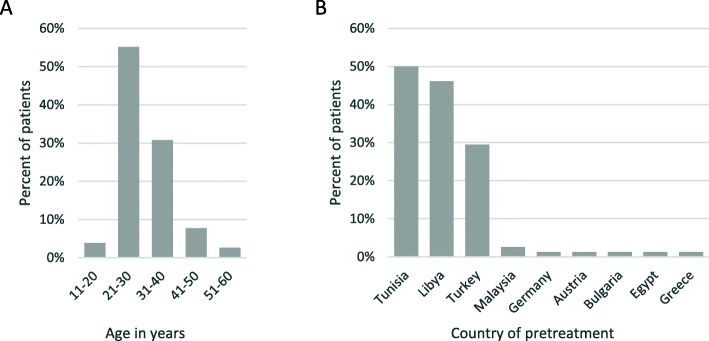
Demographics. Age in years (**a**) and country of pretreatment (**b**)

Our 78 patients (77 male and 1 female) were mainly members of militias. Patient selection was based on presentation of patients by the Libyan War Casualties’ Committee (Tripoli). Of those, the hospital committee Krankenhaus Nordwest in Germany, consisting of an experienced neurologist and an experienced orthopedic and trauma surgeon (CR and UML), decided which patients were admitted to Krankenhaus Nordwest, with regard to necessity and possibility of adequate treatment of neuropsychiatric and trauma surgical injuries. The mean age of the patients was 30.6 years, with the oldest being 51 years old and the youngest being an 11-year-old girl. Most of them received their primary and secondary care in Tunisia (*n* = 39), followed by Libya (*n* = 36) and Turkey (*n* = 23). Thirty-one patients had been treated in two or more countries before receiving definite care in Germany. Further countries of primary and secondary care were Malaysia, Austria, Bulgaria, Egypt and Greece (Fig. 1).

The peripheral and central nervous involvement was based on the findings of the clinical neurological examination, electrophysiological examination and neuroradiological examination. Detection of psychiatric manifestations was based on the psychologic evaluation of a trained psychologist. Polytrauma was defined as having an Injury Severity Score (ISS) ≥ 16. The ISS ranges from 3 to 75 and assesses trauma severity (1: minor, 2: moderate, 3: serious, 4: severe, 5: critical, 6: maximal) in 6 regions (head or neck, face, chest, abdomen/pelvic contents, extremities or pelvic girdle, external). For score calculation, the scores assessing trauma severity from the three most severely injured regions are squared and added together. Injuries treated in primary and secondary care were included in the ISS calculation. The chi-squared test was used to analyze differences in injury pattern in explosion and gunshot injuries. The significance level was set to *p* < 0.05. Statistical analysis was performed using IBM SPSS Statistics 25.

## Results

### Infrastructure preparation and management of war casualties

The Libyan War Committee (Tripoli Government) decided which patients were presented to Krankenhaus Nordwest for further evaluation. Mostly male members of militias were presented. An experienced orthopedic and trauma surgeon (CR) examined and evaluated many patients (more than 500) in Libya and Tunisia with regard to treatment necessity and possibility. Libyan medical doctors acted as interpreters and companions. Previous medical records including images of injuries of primary or secondary care hospitals were sent to our hospital. In Germany, the hospital committee Krankenhaus Nordwest (CR and UML), sifted through the files to determine whether patients were stable enough for transport and to determine possible recovery potential. Medical futility cases with no realistic chance of benefit were not selected. This enabled inpatient admissions depending on available capacities. Due to high risk of multidrug resistant organisms, a dedicated ward was prepared with isolation and hygienic protocols, including clinical examination of all patients and nasopharyngeal, inguinal, rectal and wound swabs upon admission to test patients for multidrug resistant organisms. Patients who were not bedridden, were accommodated in a hotel. Inpatients were isolated until final swab results became available and treatment of outpatients was initialized after final swab results were obtained. Depending on the findings, isolation was retained for treatment. A decontamination protocol was started in all patients at arrival and was discontinued in case of negative swab results [19, 30]. Two Libyan medical doctors were permanently available for patients’ support. Further preparation of the hospital included recruitment of interpreters and Arabic-speaking nurses as well as intercultural training of doctors, nurses and therapists. For rumor control, contact with media was kept to a minimum.

### Injury mechanisms

**Fig. 2:**
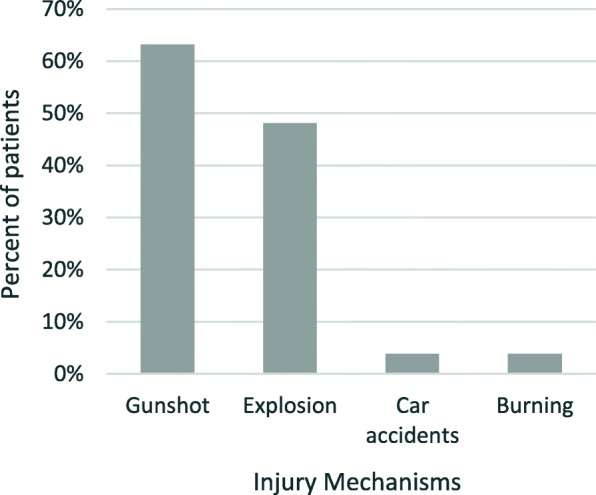
Injury Mechanisms. Frequency of gunshots, explosions, car accidents and burning

The most common injury mechanisms were gunshot injuries (*n* = 48, 63%), explosion injuries (*n* = 37, 41%), both (*n* = 11, 14%) and less frequent car accidents (*n* = 3, 4%) and burning (*n* = 3, 4%) (Fig. 2). One indirect injury mechanism included a transmitting bullet that caused a shock wave and dissection of the left carotid artery and subsequent stroke.

### Neuropsychiatric presentation

**Fig. 3:**
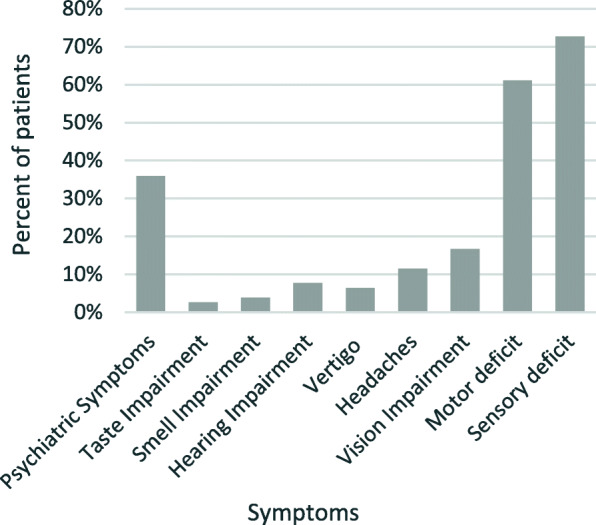
Abundance of symptoms

The patients presented with a variety of neurological deficits. Sensory (*n* = 56) and motor deficits (*n* = 47) were most common. Impairment in taste (*n* = 2), smell (*n* = 3), hearing (*n* = 6), vertigo (*n* = 5), headaches (*n* = 9) and vision impairment (*n* = 13) (Fig. 3) were reported, as well as aphasia. Seven patients were diagnosed with new onset epilepsy. Twenty-eight of our patients (36%) presented with psychiatric symptoms, including cognitive deficits, amnesia, sleep disturbance, changes in libido, avolition, affective flattening, depressed mood, mood swings or increased anxiety since the traumatic event, intrusion symptoms such as flashbacks, avoidance of trauma-related stressors with impairment of everyday life, altered arousal and reactivity, or endangerment to self and others.

### Diagnostics

The most common diagnostic procedures were neurological and orthopedic physical examinations, followed by X-rays (*n* = 46), CT scans (*n* = 33) and magnetic resonance imaging (MRI) (n = 9). Cave: As to common residing metal splinters in 30 of patients, MRI was often not possible. Neurophysiological examination included electromyography (*n* = 27) and neurography (*n* = 18). The electromyography (*n* = 27) and neurography (*n* = 18) were crucial diagnostics for detecting the severity of peripheral nerve damage. Further diagnostic methods included duplex sonography of the carotids (*n* = 11) nerve sonography (*n* = 5) and electroencephalography (*n* = 2).

### Injury patterns

**Fig. 4:**
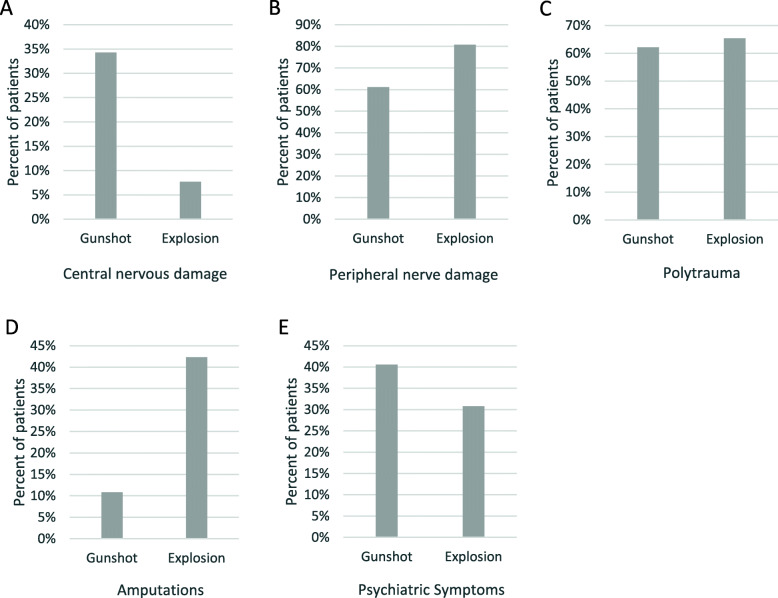
Injury patterns of gunshot and explosion injuries. Central (**a**) and peripheral (**b**) nervous damage, polytrauma (**c**), amputations (**d**) and psychiatric symptoms (**e**)

The most commonly injured areas involved the upper and lower extremities (*n* = 53), the head (*n* = 16), the thoracoabdominal region (*n* = 14) and the spine (*n* = 12). The main injuries were of neurological, orthopedic and neurosurgical nature. Damage to the central and peripheral nervous systems was diagnosed in 18 (24%) and 50 (66%) of the patients. Central nervous damage occurred significantly more in patients with only gunshot injuries (34%) than in those with only explosion injuries (8%) (*p* = 0.015) (Fig. 4). The consequences of damage to the central nervous system were severe. Thirteen of the patients had traumatic brain injury, 7 suffered from paraplegia and 4 had symptoms of hemiplegia. There was a trend association (*p* < 0.1) of peripheral nerve damage occurring more frequently in only explosion injuries (80.8%) compared to only gunshot injuries (61.1%) (Fig. 4). The ulnar (*n* = 12), radial (*n* = 11) and median (*n* = 5) nerves in upper extremity and peroneal (*n* = 21), tibial (*n* = 9) and sciatic (*n* = 6) nerves in the lower extremity were affected. Damage to the cranial nerves was diagnosed in 3 patients. One patient suffered from a lesion of the brachial plexus. Another patient suffered a stroke, most likely as a result of dissection of the left carotid artery with a vascular wall hematoma secondary to the shock wave. Psychiatric symptoms suggestive of post-traumatic stress disorder were found in 25 patients with a similar prevalence in gunshot and explosion injuries (42.4 and 31.8%, p = n.s.) (Fig. 4). Most injuries were severe, 51 suffered from polytrauma (66%), with rates in gunshot injuries and explosion injuries being similar (62.2 and 65.4%; p = n.s.) (Fig. 4). Thirty patients had residual splinters in situ. Forty-nine patients had at least one fracture (79%) and 20 had multi-fragment fractures. 18 (23%) patients were amputated before admission to our hospital. Of those, 8 (44%) presented signs of residual limb pain, while 10 (56%) complained of phantom limb pain.

### Therapy

Most common therapy was surgery in 56 cases (72%), most frequently trauma surgery (*n* = 46), followed by neurosurgery (*n* = 10) and plastic surgery (*n* = 4). Physiotherapy and structured rehabilitation programs were indicated in 49 patients to improve function after nerve and muscle damage. Fewer patients received occupational therapy and logopedic therapy. Two patients (3%) aged 23 and 27 years died after abdominal surgery as a result of their internal injuries.

## Discussion

We analyzed 78 patients with war injuries with regard to hospital preparation and management for treatment of patients injured in war, demographic variables, sex, age and country of pretreatment, injury mechanisms, symptoms, clinical diagnostics, injury patterns and therapies. We found a broad spectrum of neurological, psychiatric and trauma surgical injuries.

### Description of lessons learned

Planning regarding infrastructure, preparation and management for trauma centers worldwide for treatment of multi-cultural populations with war injuries may be needed anytime. Due to a high number of casualties that can occur in war, a preselection of patients with regard to necessity for treatment, stability for transport and a realistic chance of benefit is necessary. We achieved this through assessment of patients by independent medical doctors with expertise in trauma surgery and neuropsychiatry before admission to our hospital. As cross-border transfer of patients involves a high risk for multidrug resistant organisms [22], awareness, adequate screening and containment measures are needed. These should involve contact to a public health department and can involve the preparation of a dedicated ward, with isolation as well as hygienic protocols and repeated swabs. All procedures performed at our hospital were translated by Arabic interpreters, who had also been instructed in hygienic measurements. Interpreters as well as intercultural training have to be organized for clinical care on ward, including screening for often stigmatized psychiatric comorbidities. Rumor control and points of contact with the media should be planned in advance.

### Demographics

Our findings confirm the sex-specific bias in war injuries, with adolescent males being predominantly affected in war injuries. In literature, over 89% of patients affected are male with a mean age of 25 to 31 years in literature [2, 3, 6, 15]. The preselection of patients was based on decisions of the Libyan War Committee.

### Injury mechanisms

Our findings confirm a broad distribution of injury mechanisms [2, 11, 20, 26], with gunshots, blast injuries, car accidents and burning. One may hypothesize that the diverse pattern of injury and use of weapon is a feature of Civil War. This is different in government military action, where drones are used strategically with subsequent frequent explosion injuries [17]. Indirect injury mechanisms are common. High-velocity bullets have a large amount of kinetic energy that is transferred eccentrically from the projectile to the tissue, thereby impacting distant tissue with shock waves and creating a temporary wound cavity. This is in contrast to low-velocity bullets, which cause compression waves affecting adjacent tissue [3]. One patient suffered a stroke subsequent to a dissection of the left carotid artery, which was probably induced by a shock wave. This was most likely due to a high-velocity bullet. The broad distribution of injuries underlines the requirement of specialized trauma expertise in treatment of war victims [24, 32].

### Symptoms

Apart from the well-known neurological findings including sensory and motor deficits [6], some symptoms that we found, such as impairment of the senses (hearing, smell, taste), headache, vertigo, epilepsy and psychiatric symptoms, are rarely described in literature [31, 38] and are often overlooked when treating patients with polytrauma. These should be actively screened for in patients with war injuries. Epilepsy is an important manifestation after brain trauma. As late onset epilepsy occurs months or years after the hemorrhage and requires prolonged and often lifelong treatment, it should be screened for periodically. Psychiatric symptoms are common after experiencing war [8] and are present to a high degree in our patients. As symptoms related to trauma pose great stigma and are coped with differently in different cultural backgrounds, cultural training is essential to conduct successful diagnosis and treatment. Patients with war injuries should have a complete neurological and psychiatric assessment, as neuropsychiatric deficits are a major reason for later disability in professional and private life.

### Injury patterns neurology

Limbs are most severely affected, which has been shown in literature before [26], with a predominance of affection of the lower limbs [26]. However, there could be bias due to secondary care. Only few armies have surgical therapies available within an hour and these poorly trained fighters had no protective equipment. Therefore abdominal, chest and head injuries as well as extensive burns are poorly survived in such a war [32] and peripheral injuries are common in our patients. Furthermore, significant injuries to the central nervous system, brain injuries, spinal cord injuries and injuries to the peripheral nervous system occur [3, 15], which is supported by our data, with a high percentage of patients having peripheral nervous system affection. The most commonly affected nerves in literature as well as our patients are the radial and ulnar nerve in the upper limb and the peroneal nerve in the lower limb [6, 15]. Hence, specialized neurological and trauma surgical expertise about injuries of peripheral nerves is important.

### Injury patterns psychiatry

Psychiatric symptoms were common in our patients, displaying the importance of screening for psychiatric comorbidities. The prevalence of post-traumatic stress disorder in civil war among civilians who have experienced at least one potential traumatic event has been described to be 26.1% [27]. The prevalence increases with the number of firefights undergone [18], which could explain the high prevalence of psychiatric symptoms (36%) we found. However, our relatively young patients, who were not only defending themselves but were actively involved in combat, refused a complete assessment of a possible post-traumatic stress disorder. There was a lot of stigmata attached to psychological and psychiatric evaluation. Combat exposure and trauma to head and CNS injuries are associated with depression and anxiety, which could also explain the high rate of psychiatric symptoms [9]. Part of the older population is still affected by having experienced war as an adolescent [29], showing that early intervention is important to prevent long-term effects. Trauma affects not only the individual exposed but even the next generation, including altered foetal development and maturation factor of the brain and altered growth factors [13, 21]. Screening for psychiatric comorbidities, especially in those with close and repeated combat, is necessary for their detection as well as for consequent treatment and for prevention of further complications such as homelessness [35].

### Injury patterns trauma surgery

War injuries often induce significant damage to the human body, which frequently leads to polytrauma [2] as shown in our data. Bone injuries have been shown to have a high prevalence [26], resulting in amputations in up to 4.5% of patients within the first weeks after injury [11, 26, 28] with a high prevalence of phantom limb pain of 41–85% [1, 33]. Although we found a higher amputation rate in our patients, possibly due to preselection of patients, our findings confirm the importance of screening for complications of amputations even weeks after the surgery.

### Therapy

Appropriate surgical treatment and wound exploration of war injuries is necessary in up to 57% of cases. Fracture fixation is required in 12% of patients [11, 26]. However, our patients underwent surgical treatment more often, which shows that even in tertiary care hospitals, surgical reevaluation and treatment is necessary. Neurorehabilitation and further psychiatric evaluation and therapy was often indicated. Psychotherapy that extends until after initial patient discharge, can reduce prevalence of post-traumatic stress disorder, depression and anxiety [34] even 6 months after treatment [4]. However, the majority of our patients refused trauma-related diagnosis or therapy at the time of the study, which could be due to lack of education in our patient cohort.

Most battlefield fatalities occur on site before reaching treatment facilities [14]. The mortality rate of war injured patients in treatment facilities is variable and up to 31% in literature [3]. Our patients had to be injured with sufficient severity to require specialized treatment in Germany but had to be stable enough for transport from Libya to Germany, which could explain the low mortality rate we found in our patient group and shows that patient collectives in tertiary care sites differs substantially from the ones in primary care sites within war zones. The subjective outcome of the treatment was good in most of the patients. Obtaining an objective outcome was difficult, as most of the patients were sent back to Libya after treatment and continued rehabilitation in their home country. Follow-up appointments were frequently missed.

## Conclusions

Little is known in Germany about the management of war injuries, but infrastructure preparation is needed for management of war casualties. Among others, challenges include language barriers, infectious challenges and complex neurological deficits and psychiatric symptoms. Knowledge about demographics, injury patterns and comorbidities are required for detection and appropriate treatment and may be needed at any time. Civil war includes a broad variety of neuropsychiatric and trauma surgical injuries. Hence, specialized diagnosis, treatment and rehabilitation as well as an interdisciplinary and intercultural approach are crucial.

## Data Availability

The data that support the findings of this study are available, however restrictions apply to the availability of the data, due to lack of patient consent to transfer data to third parties. Data are however available from the authors upon reasonable request and with permission of patients.

## Abbreviations

CT: Computed tomography
EMG: Electromyography
ISS: Injury severity score
MDRO: Multidrug resistant organisms
MRI: Magnetic resonance imaging
PTSD: Post-traumatic stress disorder
UNSMIL: United Nations Supporting Mission Libya
